# Resting-state functional MRI in multicenter studies on multiple sclerosis: a report on raw data quality and functional connectivity features from the Italian Neuroimaging Network Initiative

**DOI:** 10.1007/s00415-022-11479-z

**Published:** 2022-11-09

**Authors:** Alessandro Pasquale De Rosa, Fabrizio Esposito, Paola Valsasina, Alessandro d’Ambrosio, Alvino Bisecco, Maria A. Rocca, Silvia Tommasin, Chiara Marzi, Nicola De Stefano, Marco Battaglini, Patrizia Pantano, Mario Cirillo, Gioacchino Tedeschi, Massimo Filippi, Antonio Gallo, the INNI Network, Manuela Altieri, Riccardo Borgo, Rocco Capuano, Loredana Storelli, Elisabetta Pagani, Mauro Sibilia, Claudia Piervincenzi, Serena Ruggieri, Nikolaos Petsas, Rosa Cortese, Maria Laura Stromillo

**Affiliations:** 1 Department of Advanced Medical and Surgical Sciences, University of Campania “Luigi Vanvitelli”, Piazza Luigi Miraglia, 2, 80138 Naples, Italy; 2 Neuroimaging Research Unit, Division of Neuroscience, IRCCS San Raffaele Scientific Institute, Via Olgettina 60, 20132 Milan, Italy; 3 Neurology Unit, IRCCS San Raffaele Scientific Institute, Via Olgettina 60, 20132 Milan, Italy; 4 Neurorehabilitation Unit, IRCCS San Raffaele Scientific Institute, Via Olgettina 60, 20132 Milan, Italy; 5 Neurophysiology Service, IRCCS San Raffaele Scientific Institute, Via Olgettina 60, 20132 Milan, Italy; 6 Vita-Salute San Raffaele University, Via Olgettina 58, 20132 Milan, Italy; 7 Department of Human Neurosciences, Sapienza University of Rome, Viale Dell’Università, 30, 00185 Rome, Italy; 8 Institute of Applied Physics “Nello Cararra” (IFAC), National Research Council (CNR), Via Madonna del Piano, 10, Sesto Fiorentino, 50019 Florence, Italy; 9 Department of Medicine, Surgery and Neuroscience, University of Siena, Siena, Italy

**Keywords:** Multiple sclerosis, MS, Quality control, MRI, Functional magnetic resonance imaging, Functional connectivity

## Abstract

The Italian Neuroimaging Network Initiative (INNI) is an expanding repository of brain MRI data from multiple sclerosis (MS) patients recruited at four Italian MRI research sites. We describe the raw data quality of resting-state functional MRI (RS-fMRI) time-series in INNI and the inter-site variability in functional connectivity (FC) features after unified automated data preprocessing. MRI datasets from 489 MS patients and 246 healthy control (HC) subjects were retrieved from the INNI database. Raw data quality metrics included temporal signal-to-noise ratio (tSNR), spatial smoothness (FWHM), framewise displacement (FD), and differential variation in signals (DVARS). Automated preprocessing integrated white-matter lesion segmentation (SAMSEG) into a standard fMRI pipeline (fMRIPrep). FC features were calculated on pre-processed data and harmonized between sites (Combat) prior to assessing general MS-related alterations. Across centers (both groups), median tSNR and FWHM ranged from 47 to 84 and from 2.0 to 2.5, and median FD and DVARS ranged from 0.08 to 0.24 and from 1.06 to 1.22. After preprocessing, only global FC-related features were significantly correlated with FD or DVARS. Across large-scale networks, age/sex/FD-adjusted and harmonized FC features exhibited both inter-site and site-specific inter-group effects. Significant general reductions were obtained for somatomotor and limbic networks in MS patients (vs. HC). The implemented procedures provide technical information on raw data quality and outcome of fully automated preprocessing that might serve as reference in future RS-fMRI studies within INNI. The unified pipeline introduced little bias across sites and appears suitable for multisite FC analyses on harmonized network estimates.

## Introduction

Multiple sclerosis (MS) is the most frequent chronic inflammatory, demyelinating and neurodegenerative disease affecting the central nervous system (CNS) in young adults [1]. MS is a heterogeneous, multifactorial, immunological disease characterized by recurrent clinical manifestations and progression of disability over time [1]. The principal hallmark of MS is the accumulation of focal demyelinating lesions in the white and gray matter of the brain as well as in the spinal cord [1]. The diagnosis of MS is based on proof of disease dissemination in space and time and exclusion of other disorders that can mimic this condition. Thanks to its sensitivity to MS-related focal abnormalities, conventional brain MRI, including T1- and T2-weighted sequences, has become fundamental for an early and accurate diagnosis as well as for monitoring MS disease activity and response to treatment [2, 3]. In addition to its clinical value, advanced MRI techniques are improving our understanding of the structural and functional changes underlying MS pathophysiology, providing invaluable insights into disease mechanisms [4, 5]. Despite the important findings provided by MRI studies so far, there are several drawbacks, including, for most of them, the small-sample size of patients enrolled. This limitation strongly affects the robustness and reproducibility of the results obtained in the field. In contrast, studies on larger cohorts of patients enable the testing of hypotheses with superior statistical power, thus enhancing the reliability of the results [6].

In recent years, major technological advances in the analysis of neuroimages have been made thanks to the availability of large, shared neuroimaging data repositories, giving access to thousands of MRI scans on the web [7]. Several types of data sharing have been proposed and utilized. The most common form of data sharing involves data previously reported in publications in the form of coordinate-based metadata [8]. Some repositories contain already-processed data, such as statistical maps [9], while others contain raw datasets from individual subjects [10, 11]. Notable examples of established sharing initiatives collecting MRI data from patients and healthy subjects are the Alzheimer’s Disease Neuroimaging Initiative (ADNI), the Autism Brain Imaging Data Exchange (ABIDE), and the UK Biobank [12–15].

The Italian Neuroimaging Network Initiative (INNI) has supported the creation of a centralized repository, where brain MRI, demographical, clinical, and neuropsychological data from MS patients and healthy controls are collected from the participating sites, with the main goal of defining the role of clinical and conventional MRI biomarkers in understanding MS pathophysiology [16]. In addition, the INNI initiative will promote the use at a national level of advanced structural and functional MRI techniques to be applied for advanced studies on MS. The MRI data collected within the INNI database include high resolution 3D T1-weighted scans for anatomical volumetric studies, T2-weighted or Fluid Attenuated Inversion Recovery (FLAIR) scans for MS lesions quantification, and diffusion tensor imaging (DTI) and resting-state functional MRI (RS-fMRI) series for advanced MRI studies.

An important issue regarding the collection of large-scale multicenter MRI data is quality control (QC), as poorly acquired data can compromise the trustworthiness and reliability of a study [17]. For instance, many automated preprocessing steps (such as segmentation and registration) and the subsequent statistical inferences are highly sensitive to the presence of image artifacts and to spurious signal fluctuations [18, 19]. Moreover, the heterogeneity of scanners and acquisition protocols can potentially affect the consistency of MRI-derived features and, therefore, undermine statistical testing and/or classification performances [20, 21]. For this reason, harmonizing the MRI data is generally considered important [22]–[24] and specific recommendations for MS multicenter studies have been recently updated [25].

As regards INNI, a study was previously published to provide information on the quality of conventional and volumetric 3D T1-weighted MRI data uploaded into the repository [26].

The aim of this study is twofold. First, to propose quantitative and objective metrics that can characterize the quality of brain RS-fMRI raw datasets from single MS patients collected within the INNI repository. Second, to assess the variability of functional MRI quality and connectivity features across different sites and scanners when the former is assessed on raw data and the same preprocessing pipeline is applied to obtain the latter, as well as the relation between quality metrics and features. Based on the results, the INNI consortium could eventually promote a standardized use of these quality metrics to compare the results of functional connectivity (FC) studies on the same multicenter MRI dataset.

## Materials and methods

The INNI project is promoted by the Neuroimaging Study Group of the Italian Society of Neurology and is financially supported by a special research grant from the Fondazione Italiana Sclerosi Multipla (FISM). FISM is the owner of the database, according to Italian copyright law. The INNI currently involves four Italian MS centers (Neuroimaging Research Unit, Division of Neuroscience, IRCCS San Raffaele Scientific Institute; Department of Human Neuroscience, “La Sapienza” University, Rome; Department of Neurological Sciences, University of Campania Luigi Vanvitelli and Care “Hermitage Capodimonte”, Naples; Department of Neurological and Behavioural Sciences, University of Siena, Siena).

### Datasets

MRI data from MS patients and healthy control (HC) subjects were retrieved from the INNI repository (https://database.inni-ms.org) based on the current availability of at least one anatomical 3D-T1-weighted (3D-T1w) scan and one RS-fMRI scan from the same exam. Based on the demographic data (age and sex) of the MS patients, HCs were selected to maximize the size of a sample of HCs age- and sex-matched to the MS sample for each center. As a result, MRI data from the exams of 489 MS patients with the main clinical MS phenotypes (relapsing–remitting [RRMS], primary progressive [PPMS], secondary progressive [SPMS], benign [BMS] and clinically isolated syndrome [CIS]), and 246 HCs acquired in all four participating centers (labeled here as A, B, C and D), were downloaded. All MRI exams were executed on 3 Tesla scanners: Intera and Achieva, respectively, for Centers A and D (Philips Medical Systems, Best, The Netherlands); Signa HDxt for Center B (GE Healthcare, Milwaukee, USA); Magnetom Verio for Center C (Siemens, Erlangen, Germany). Patient positioning was performed according to the usual internal procedures of each center. In each center, all MRI data were acquired on the same scanner and with the same protocol, except for Center A, where 36 MS patients and 17 HCs scans were acquired with the same anatomical sequence but with slightly different acquisition parameters. Demographic and clinical data and MRI acquisition parameters for each center are summarized, respectively, in Tables 1 and 2.

**Table 1:** Demographic and clinical information for each center participating in the INNI initiative (the statistically significant differences are reported in bold)

	Center A	Center B	Center C	Center D	Total
Patients/controls	260/99	141/94	72/37	16/16	489/246
Age, mean ± sd					
Patients	40.9 ± 12.3	37.1 ± 10.3	40.3 ± 11.0	42.7 ± 7.18	39.8 ± 11.6
Controls	38.1 ± 13.5	38.2 ± 11.6	37.2 ± 12.8	42.6 ± 6.97	38.3 ± 12.4
*p* (*t* value)	0.08 (1.78)	0.45 (− 0.76)	0.21 (1.27)	0.98 (0.02)	0.12 (1.55)
Sex					
Patients	156 F/104 M	94 F/47 M	54 F/18 M	12 F/4 M	316 F/173 M
Controls	51 F/48 M	54 F/40 M	23 F/14 M	12 F/4 M	140F/106 M
*p* (${\chi }^{2}$ value)	0.15 (2.11)	0.15 (2.06)	0.16 (1.94)	1.00 (0.00)	**0.04 (4.13)**
MS phenotype (RR/SP/PP/CIS/B)	166/53/18/0/23	122/4/1/14/0	65/3/1/3/0	15/0/0/0/1	368/60/20/17/24
Disease duration, mean ± sd	12.7 ± 8.54	11.1 ± 8.98	9.57 ± 6.52	8.56 ± 9.14	11.9 ± 8.52
EDSS, median (IQR)	2 (3)	2 (1.5)	1.5 (1.5)	1.5 (0.5)	2 (2.5)
SDMT, mean ± sd	42.5 ± 16.0	40.3 ± 14.7	45.2 ± 11.4	45.8 ± 13.4	42.4 ± 15.0

**Table 2:** MRI acquisition parameters for each center participating in the INNI initiative

	Center A	Group B	Group C	Group D
Scanner	Philips Intera	GE Signa HDxt	Siemens Verio	Philips Achieva
Coil	8-channel coil	8-channel coil	12-channel coil	32-channel coil
3D-T1 weighted				
Sequence	[FFE–TFE]	IR-FSPGR	MPRAGE	TFE
Imaging plane	[Axial–Sagittal]	Sagittal	Sagittal	Axial
Spatial resolution [mm^3^]	[0.9 × 0.9 × 1.6–1 × 1 × 1]	1 × 1 × 1.2	0.5 × 0.5 × 1	1 × 1 × 2
Acquisition matrix	[256 × 256–256 × 256]	256 × 256	256 × 256	256 × 256
Slices	[220–204]	166	176	192
TR [ms]	[25–7]	6.9	1900	10
TE [ms]	[4.6–3.1]	2.8	2.9	4
TI [ms]	–	650	900	–
Flip angle [°]	[30–9]	8	[8–9]	8
RS-fMRI				
Sequence	EPI	EPI	EPI	EPI
Imaging plane	Axial	Axial	Axial	Axial
Spatial resolution [mm^3^]	1.875 × 1.875 × 4	4 × 4 × 4	3 × 3 × 3	1.875 × 1.875 × 4
Acquisition matrix	128 × 128	64 × 64	64 × 64	128 × 128
Slices	30	29	50	30
TR [ms]	3000	1500	3000	3000
TE [ms]	35	32	30	35
Flip angle [°]	90	90	89	90
Volumes	200	240	140	200

Study approval was obtained from the local ethics committee of each participating center and written informed consent was given by all participants at the time of data acquisition.

### Image quality metrics

We used the MRIQC workflow [27] to derive a set of image quality metrics (IQMs) to assess the quality of the raw RS-fMRI data. A justification for the use of these IQMs and a complete description of the metrics can be found at https://mriqc.readthedocs.io/en/latest/measures.html. For the sake of simplicity, we selected only a subset of the IQMs provided by MRIQC: temporal signal-to-noise ratio (tSNR) was selected as a reliable measure for temporal information; full width at half maximum (FWHM) was preferred to spatial signal-to-noise ratio (SNR) as measure for spatial information, since the use of multichannel phase-array surface coils and parallel imaging acquisition techniques would not provide an accurate assessment of the spatial SNR [28]; framewise displacement (FD) [29] is the most common measure to summarize head motion; differential variation in the signal (DVARS) [30, 31] is another popular measure to characterize fMRI data quality with respect to the contribution of non-neural sources (including also physiological noise).

The tSNR is calculated in each voxel as the ratio between the average BOLD signal across time and the corresponding temporal standard deviation [32]. Higher tSNR values are indicative of a superior ability to detect small changes in image intensity associated with subtle brain activation [33]. The FWHM describes the average width of local extrema in the intensity of an image. The mean FWHM (over time) is indicative of the average intrinsic smoothness of the images (prior to preprocessing). Higher mean FWHM values increase the ability to detect small BOLD signal changes over contiguous pixels [34]. DVARS indexes the rate of change of BOLD signal across the entire brain at each time point and higher values for mean DVARS indicate higher levels of physiological noise. Here, we used a standardized version for DVARS, where the mean values are normalized to the standard deviation of the temporal difference time series. For a given image time-series, the mean FD is indicative of the average amount of motion, regardless of the type (translation or rotation) and orientation. Higher values for mean FD indicate higher levels of head motion (prior to preprocessing). All IQMs were evaluated prior the subsequent MRI data preprocessing, which was carried out after the exclusion of high-motion subjects (i.e., mean FD > 0.25 mm) [35].

### Structural MRI preprocessing

All anatomical scans were resampled to an isometric 1 × 1 × 1 mm grid to standardize the structural MRI preprocessing. Automatic brain tissue segmentation was performed with FreeSurfer v7.1.1 [36]. We used the Sequence Adaptive Multimodal SEGmentation (SAMSEG) [37] procedure to automatically and simultaneously perform whole-brain tissue segmentation (including white matter [WM], gray matter [GM], cerebrospinal fluid [CSF] segmentation), and MS lesion segmentation. The characteristic property of SAMSEG is that it accepts multi-contrast MRI data without specific requirements on the pulse sequences (e.g., 2D or 3D) and is therefore well suited for multicenter MS studies. Nonetheless, we segmented each anatomical data set using only the 3D T1-weighted images, as FLAIR scans were not available from all sites.

### Functional MRI preprocessing

Functional MRI preprocessing was carried out using fMRIPrep v20.2.1 [38]. The following steps were applied to all datasets: skull stripping, motion correction, slice time correction, susceptibility distortion correction, and co-registration of the functional and anatomical scans. Of note: as slice acquisition timing information was missing in the DICOM files, the slice time correction was skipped for Philips datasets. Moreover, as field maps or reverse phase encoding acquisitions were not available for most of the exams, fMRIPrep adopted a field map-less susceptibility distortion correction procedure which is based on nonlinear registration of the EPI images to the same-subject T1w images [39].

Automatic removal of motion artifacts using independent component analysis ICA-AROMA [40] was performed on the pre-processed BOLD time-series. ICA-AROMA motion components were collected and used as noise regressors along with the mean physiological noise signals from WM and CSF. Finally, the time series were band-pass filtered between 0.01 Hz and 0.1 Hz.

### Functional connectivity features

For each participant, FC network features and (within-GM) global connectivity features were calculated (see Sects. 2.5.1 and 2.5.2). Prior to the statistical analyses, all features were adjusted for age, sex, and mean FD within each center using linear regression. The entire preprocessing pipeline and the extraction of the features was carried out on a HP Z6 G4 workstation equipped with two 8-core Intel (R) Xeon (R) Bronze 3106 @1.70 GHz, for a total of 16 CPU threads and 128 GB RAM. For each data set (from one patient), the total processing time (on a single CPU core) was approximately 4 h.

#### Network-level features

The mean time-series were extracted from 100 pre-defined cortical parcels in native space using the Schaefer atlas [41]. These regions are clustered into seven functional networks: visual (VN), somatomotor (SMN), dorsal attention (DAN), ventral attention (VAN), limbic (LN), frontoparietal (FPN), and default (DMN) [42]. The Pearson correlation coefficient was computed between the mean time-series of each pair of regions, in a 100 × 100 connectivity matrix for each participant. Fisher’s *r*-to-*z* transformation was applied for all connectivity matrices to improve the normality. For each network, the average correlation coefficient was calculated from the within-network positive values, as the correct interpretation of negative correlations is notoriously ambiguous [43–45].

#### Global features

Voxel-based maps of intrinsic brain activity and connectivity were computed using the Data Processing and Analysis of Brain Imaging (DPABI) toolbox [46] in MATLAB R2021a (The MathWorks Inc., Natick, Massachusetts, United States). Amplitude of low-frequency fluctuations (ALFF) was taken as a voxel-based measure of the amplitude of spontaneous neural signal fluctuations from the filtering of the BOLD signal in the frequency range of spontaneous neural activity (0.01–0.1 Hz) [47]. ALFF maps were extracted from the time-series prior to band-pass filtering. Regional homogeneity (ReHo) is based on the local synchronization between the time series of a given voxel and its nearest 26 neighboring voxels and was taken as another voxel-based measure of brain activity [48]. A higher ReHo value for a given voxel indicates higher regional coherence. Finally, degree centrality (DC) was taken as a measure of local–global FC; it is defined as the number of voxels across the whole brain that show strong correlation (above a threshold of *r* > 0.25) with a target voxel [49]. Following the work from Buckner et al., we chose a threshold of 0.25 to remove connections that had low temporal correlation attributable to noise (in the same work, the authors showed that different thresholds did not qualitatively change the results). From a brain network perspective, DC is measured as the number of connections a voxel has with the rest of the brain (binarized DC), or the sum of weights across all those connections (weighted DC) [50]. We used weighted DC, since it provides a more reliable measure for characterizing centrality of functional brain networks [51]. RS-fMRI maps were extracted as reported in [52]: ALFF maps were calculated in native space and then normalized to the Montreal Neurological Institute (MNI152) template; instead, following the work from Yan et al., ReHo and DC maps were directly calculated in MNI152 space to ensure the same voxel size across sites [52]. All maps were Z-standardized and further smoothed with a Gaussian kernel of 4.5 mm [52]. For each of these features, we computed the mean across the individual GM mask, thus summarizing the global brain activity and connectivity across the entire GM.

### Feature harmonization

Harmonization techniques are used to handle non-biological variance introduced by differences in MRI scanners and acquisition protocols [25]. To remove these unwanted effects, we applied the ComBat harmonization [53, 54] in its R implementation (freely available at https://github.com/Jfortin1/ComBatHarmonization) to all datasets. We included age, sex, and group (controls: 0; MS: 1) as biological covariates in the harmonization process to preserve them from the removal of scanner-related effects in the presence of different age and sex distributions between sites. However, harmonized features were subsequently adjusted for age, sex, and mean FD prior to the multisite statistical analysis.

### Statistical analysis

Statistical analysis was performed with the R software version 4.1.2 (R Foundation for Statistical Computing, Vienna, Austria. URL: https://www.R-project.org/). The Pearson’s chi-squared test (${\chi }^{2}$ test) was used to test for differences in the MS phenotype and sex group distributions among the four participating centers; the analysis of variance (ANOVA) and the Student’s *t* test were used to test for differences in age among the four participating centers and between centers. Kruskal–Wallis tests due to non-normal distribution of data (identified using the Shapiro–Wilk test) were used to evaluate differences in IQMs and FC features among the different centers. For post hoc testing, Dunn’s test was used to determine which centers produced estimates that were significantly different from others. Bonferroni correction was applied to adjust *p* values for multiple comparisons. A *p* value < 0.05 was considered as a statistically significant result.

Because the scale of the measurements could be also affected by inter-site differences [53], the variance of the FC estimates was also compared between sites using the Fligner–Killeen test to determine whether the variances are homogenous between sites [55]. In this case, we only considered the HCs of each site, as MS pathology would likely affect the variance of a group differently across sites also due to phenotypic differences.

Wilcoxon rank sum test was used to evaluate differences between MS and HCs on FC features, without and with ComBat harmonization. The correlations between FD or DVARS (from raw data) and connectivity features (from pre-processed data) were also assessed for each site to evaluate the impact of the initial technical quality of the raw data on FC features. As the role of the preprocessing should be to reduce as much as possible the influence of technical factors and artifacts on the inter-individual variability of the final features, this analysis allows to assess the impact of the initial raw data quality on the FC features when these are derived from the same preprocessing pipeline applied to the data from each center. Correlation coefficients were considered statistically significant when the *p* value of the test was below 0.05, after Bonferroni correction for all tests performed.

## Results

In this section, we illustrate the differences in demographic data and IQMs (prior to data preprocessing) across centers and scanners, along with boxplots and raincloud plots to qualitatively show the inter-site effects. Next, we illustrate the differences in the FC features (after data preprocessing) across the centers and between groups, along with boxplots and raincloud plots to qualitatively show the inter-site effects, without and with multisite harmonization applied to the features. Numerical results are reported as median values and interquartile ranges.

**Fig. 1:**
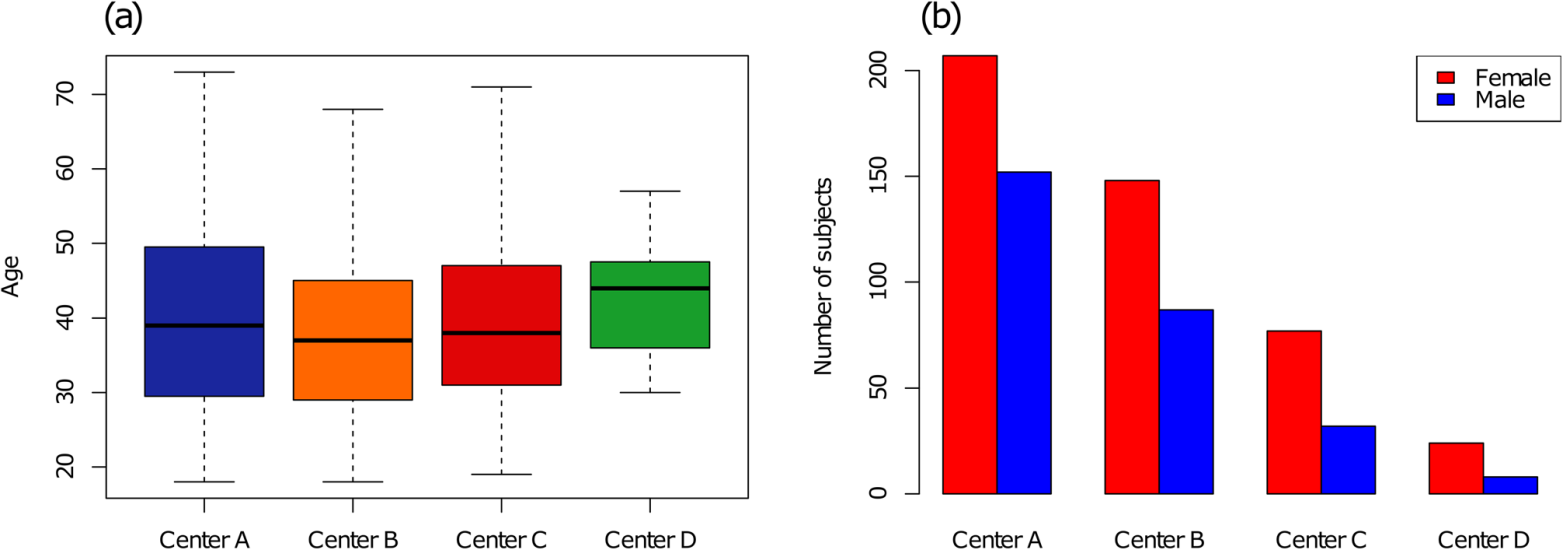
Distributions of participant’s demographical characteristics across the four centers: age (**a**) and sex (**b**)

The distribution of demographic information (age and sex) across sites is shown in Fig. 1. Both the age and sex distribution were slightly unbalanced across sites (age: *p* = 0.02, sex: *p* = 0.03) albeit post hoc testing failed to reveal any significant differences for pair-wise comparisons after correction for multiple comparisons. For the MS groups, clinical MS phenotypes’ distributions were found to be unbalanced between centers (*p* < 0.001). Tissue and lesion volumes derived from SAMSEG are reported in Table 3.

**Table 3:** Tissue volumes and lesion load as derived from SAMSEG; all volumes are in units of ml and are not adjusted for age, sex, and total intracranial volumes

	Center A	Center B	Center C	Center D	
Gray matter					
Patients (MS)	437 ± 57	466 ± 51	454 ± 45	451 ± 51	
Controls (HCs)	476 ± 54	489 ± 51	482 ± 48	480 ± 51	
*p* (*t* value)	6.4 $\times$ 10^–9^ (− 6.09)	5.5 $\times$ 10^–4^ (− 3.51)	5.3 $\times$ 10^–3^ (− 2.88)	0.087 (− 1.77)	
White matter					$\times$
Patients	406 ± 52	370 ± 36	390 ± 43	375 ± 39	
Controls	423 ± 51	382 ± 41	401 ± 39	396 ± 47	
*p* (*t* value)	5.4 $\times$ 10^–3^ (− 2.81)	0.018 (− 2.38)	0.20 (− 1.30)	0.18 (− 1.36)	
Cerebrospinal fluid					
Patients	392 ± 62	408 ± 48	402 ± 52	411 ± 50	
Controls	373 ± 48	392 ± 46	392 ± 44	392 ± 48	
*p* (*t* value)	2.2 $\times$ 10^–3^ (3.10)	0.011 (− 2.55)	0.31 (1.02)	0.28 (1.10)	
Lesion load	5.18 ± 7.24	4.38 ± 6.29	3.60 ± 4.89	4.71 ± 4.42	

**Fig. 2:**
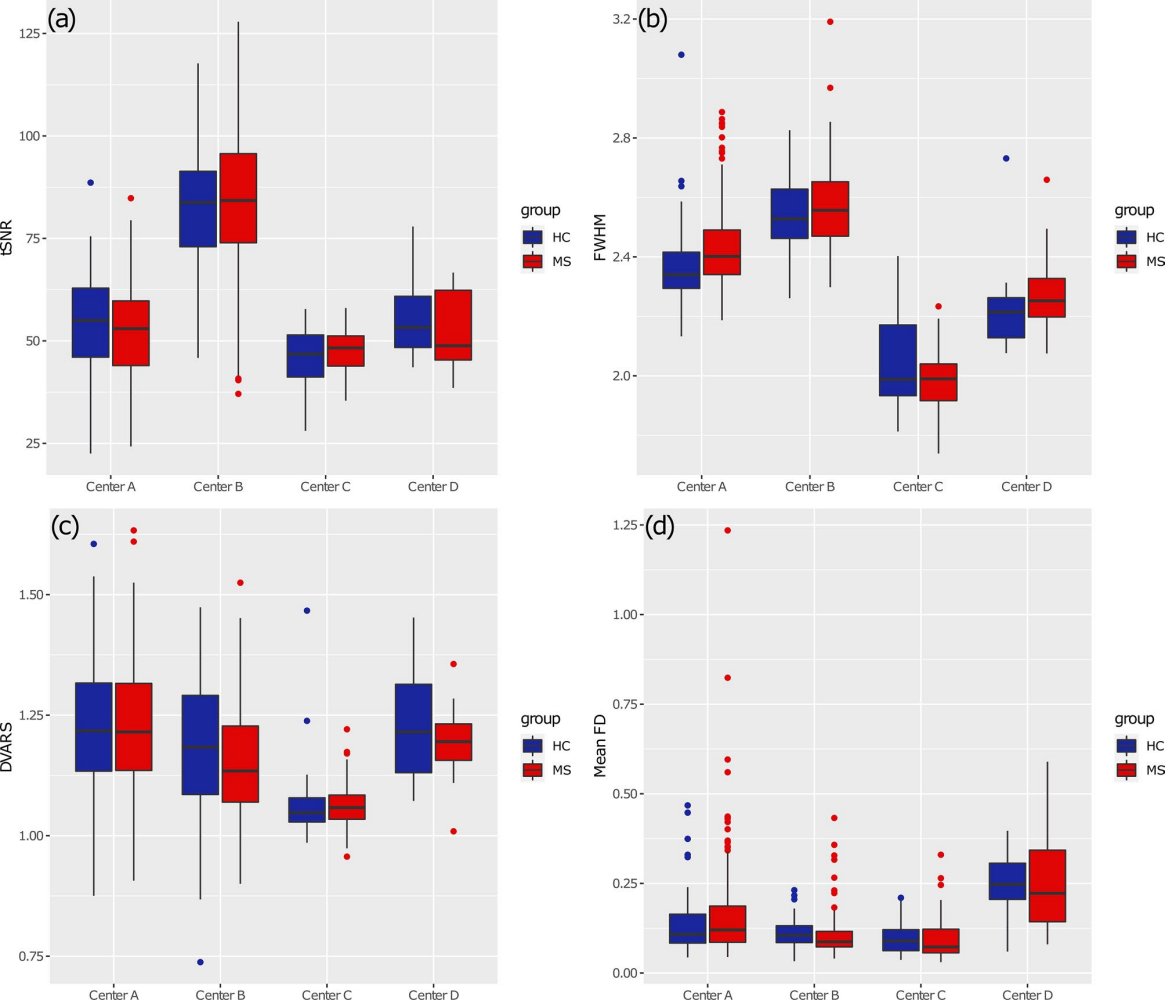
Boxplots representing IQMs for every subject’s fMRI data for different centers: median tSNR (**a**), mean FWHM (**b**), DVARS (**c**), and mean FD (**d**)

The distribution of the median tSNR across groups and sites is shown in Fig. 2a. There was a significant inter-site effect (*p* < 0.001) in the tSNR, the highest tSNR values being observed for patients and controls in Center B (median = 84.2 [16.8]; median = 83.8 [14.5]) and the lowest tSNR values being observed in Center C (median = 48.3 [5.68]; median = 46.8 [7.09]). No differences between MS patients and HCs were found in any of the centers.

The distribution of the mean FWHM across groups and sites is shown in Fig. 2b. There was a significant inter-site effect (*p* < 0.001), the highest FWHM values being observed for patients and controls acquired in Center B (median = 2.56 [0.14] mm; median = 2.53 [0.12] mm) and the lowest FWHM values being observed in Center C (median = 1.99 [0.11] mm; median = 1.99 [0.15] mm). Only for Center A, FWHM was significantly different (*p* < 0.01) between MS patients and HCs.

The distribution of the DVARS across groups and sites is shown in Fig. 2c. There was a significant inter-site effect (*p* < 0.001), the highest DVARS values being observed for patients and controls acquired in Center A (median = 1.22 [0.18]; median = 1.22 [0.18]) and the lowest DVARS values being observed in Center C (median = 1.06 [0.05]; median = 1.05 [0.05]). No differences between MS patients and HCs were found in any of the centers.

The distribution of the mean FD across groups and sites is shown in Fig. 2d. There was a significant inter-site effect (*p* < 0.001), the highest motion being observed for data acquired in Center D (median = 0.22 [0.20] mm; median = 0.25 [0.10] mm) and the lowest motion being observed for data acquired in Center C (median = 0.07 [0.06]; median = 0.09 [0.05]). No differences in mean FD between MS and HC groups were found significant in any of the centers.

Based on the motion estimates, “high-motion” subjects (i.e., subjects with mean FD > 0.25 mm) were excluded from the subsequent analyses of FC estimates from the pre-processed data. Thus, 32/260 MS and 5/99 HC from Center A, 5/141 MS from Center B, 2/72 MS from Center C, 6/16 MS and 8/16 HC from Center D were excluded, the total number of “low-motion” subjects available for the group analysis resulting in 444 MS patients and 233 HC subjects.

After exclusion of high-motion subjects (mean FD > 0.25), the age- and sex-adjusted FC estimates (as obtained from pre-processed data) were correlated with the mean FD and DVARS on a site basis. The correlation (r score with p-value) and determination (*R*^2^ score) coefficients are reported in Tables 4 and 5. According to this analysis, visual network FC was significantly (positively) correlated to DVARS in Center A (*r* = 0.19, *R*^2^ = 0.035, *p* < 0.05 after correction for multiple comparisons). Global features were significantly (positively) correlated to DVARS for the data from Center A (with the exclusion of ALFF) and from Center B; otherwise, the variance in the FC features explained by DVARS in the pre-processed data was always below 5% for all sites with the only further exception of ALFF in Center C and Center D, where four out of seven networks (SMN,VAN, LN, and DMN) displayed an R^2^ above 0.05, albeit none of these correlations were statistically significant after correction for multiple correction.. Two global features (ALFF and ReHo) were significantly (negatively) correlated to the mean FD for the data from Center B (*r* > 0.25, *R*^2^ > 0.05, *p* < 0.05 after correction for multiple comparisons); otherwise, the variance in the FC features explained by the residual motion in the pre-processed data was always below 5% for all sites with the only further exception of Center D, where three out of seven networks (SMN, LN, and FPN) and all three global features (ALFF, ReHo, and DC) displayed an *R*^2^ above 0.05, albeit none of these correlations were statistically significant after correction for multiple correction. Anyway, for the subsequent inter-site and group-level analyses, all FC features (without or with preliminary inter-site harmonization) were further adjusted for mean FD on a site basis.

**Table 4:** Correlation coefficients and *R*^2^ between DVARS and FC features (both networks and global); uncorrected *p* values are indicated in brackets

	Center A	Center B	Center C	Center D
VN	*r* = 0.19 (< 0.01) *R*^2^ = 0.035	*r* = − 0.05 (0.43) *R*^2^ = 0.003	*r* = − 0.21 (0.03) *R*^2^ = 0.044	*r* = − 0.15 (0.56) *R*^2^ = 0.022
SMN	*r* = 0.08 (0.13) *R*^2^ = 0.007	*r* = 0.05 (0.47) *R*^2^ = 0.002	*r* = − 0.06 (0.57) *R*^2^ = 0.003	*r* = 0.38 (0.12) *R*^2^ = 0.146
DAN	*r* = 0.08 (0.18) *R*^2^ = 0.006	*r* = 0.15 (0.03) *R*^2^ = 0.022	*r* = − 0.04 (0.69) *R*^2^ = 0.002	*r* = 0.01 (0.97) *R*^2^ = 0.0001
VAN	*r* = 0.16 (< 0.01) *R*^2^ = 0.024	*r* = 0.14 (0.04) *R*^2^ = 0.018	*r* = − 0.01 (0.90) *R*^2^ = 0.0002	*r* = 0.52 (0.03) *R*^2^ = 0.266
LN	*r* = 0.03 (0.65) *R*^2^ = 0.001	*r* = − 0.13 (0.05) *R*^2^ = 0.017	*r* = − 0.11 (0.24) *R*^2^ = 0.013	*r* = 0.41 (0.09) *R*^2^ = 0.171
FPN	*r* = 0.05 (0.40) *R*^2^ = 0.002	*r* = 0.07 (0.27) *R*^2^ = 0.005	*r* = 0.08 (0.41) *R*^2^ = 0.007	*r* = 0.01 (0.96) *R*^2^ = 0.0001
DMN	*r* = 0.10 (0.07) *R*^2^ = 0.010	*r* = 0.07 (0.26) *R*^2^ = 0.005	*r* = 0.09 (0.33) *R*^2^ = 0.009	*r* = 0.23 (0.36) *R*^2^ = 0.052
ALFF	*r* = − 0.06 (0.30) *R*^2^ = 0.003	***r* = 0.28 (< 0.01)** ***R***^**2**^** = 0.078**	*r* = − 0.24 (0.01) *R*^2^ = 0.056	*r* = 0.21 (0.40) *R*^2^ = 0.044
ReHo	***r* = 0.28 (< 0.01)** ***R***^**2**^** = 0.080**	***r* = 0.31 (< 0.01)** ***R***^**2**^** = 0.096**	*r* = − 0.08 (0.39) *R*^2^ = 0.007	*r* = 0.20 (0.42) *R*^2^ = 0.040
DC	***r* = 0.21 (< 0.01)** ***R***^**2**^** = 0.044**	***r* = 0.23 (< 0.01)** ***R***^**2**^** = 0.053**	*r* = − 0.04 (0.68) *R*^2^ = 0.002	*r* = 0.14 (0.59) *R*^2^ = 0.019

Values in bold indicate statistically significant correlations after Bonferroni correction (*p* < 0.05)

**Table 5:** Correlation coefficients and *R*^2^ between Framewise Displacement (FD) and FC features (both networks and global); uncorrected *p* values are indicated in brackets

	Center A	Center B	Center C	Center D
VN	*r* = 0.03 (0.57) *R*^2^ = 0.001	*r* = − 0.01 (0.89) *R*^2^ = 0.0001	*r* = 0.09 (0.34) *R*^2^ = 0.009	*r* = 0.09 (0.72) *R*^2^ = 0.008
SMN	*r* = − 0.11 (0.04) *R*^2^ = 0.012	*r* = − 0.06 (0.38) *R*^2^ = 0.003	*r* = − 0.15 (0.13) *R*^2^ = 0.021	*r* = − 0.57 (0.01) *R*^2^ = 0.327
DAN	*r* = − 0.10 (0.08) *R*^2^ = 0.010	*r* = − 0.10 (0.15) *R*^2^ = 0.009	*r* = − 0.19 (0.04) *R*^2^ = 0.037	*r* = − 0.14 (0.59) *R*^2^ = 0.019
VAN	*r* = − 0.13 (0.02) *R*^2^ = 0.017	*r* = − 0.11 (0.10) *R*^2^ = 0.012	*r* = − 0.07 (0.48) *R*^2^ = 0.005	*r* = − 0.02 (0.94) *R*^2^ = 0.0003
LN	*r* = − 0.12 (0.03) *R*^2^ = 0.015	*r* = − 0.04 (0.59) *R*^2^ = 0.001	*r* = − 0.12 (0.23) *R*^2^ = 0.013	*r* = − 0.27 (0.28) *R*^2^ = 0.071
FPN	*r* = 0.02 (0.78) *R*^2^ = 0.00023	*r* = 0.06 (0.38) *R*^2^ = 0.003	*r* = − 0.04 (0.69) *R*^2^ = 0.002	*r* = 0.35 (0.16) *R*^2^ = 0.121
DMN	*r* = − 0.09 (0.09) *R*^2^ = 0.009	*r* = − 0.01 (0.88) *R*^2^ = 0.0001	*r* = − 0.03 (0.73) *R*^2^ = 0.001	*r* = 0.07 (0.77) *R*^2^ = 0.006
ALFF	*r* = − 0.14 (0.01) *R*^2^ = 0.020	***r* = − 0.27 (< 0.01)** ***R***^**2**^** = 0.074**	*r* = − 0.15 (0.12) *R*^2^ = 0.023	*r* = 0.26 (0.29) *R*^2^ = 0.070
ReHo	*r* = − * r* = − 0.15 (0.01) *R*^2^ = 0.024	***r* = − 0.28 (< 0.01)** ***R***^**2**^** = 0.077**	*r* = − 0.14 (0.14) *R*^2^ = 0.021	*r* = − 0.23 (0.36) *R*^2^ = 0.052
DC	*r* = − 0.08 (0.16) *R*^2^ = 0.006	*r* = − 0.11 (0.08) *R*^2^ = 0.013	*r* = − 0.13 (0.18) *R*^2^ = 0.017	*r* = − 0.23 (0.36) *R*^2^ = 0.053

Values in bold indicate statistically significant correlations after Bonferroni correction (*p* < 0.05)

**Fig. 3:**
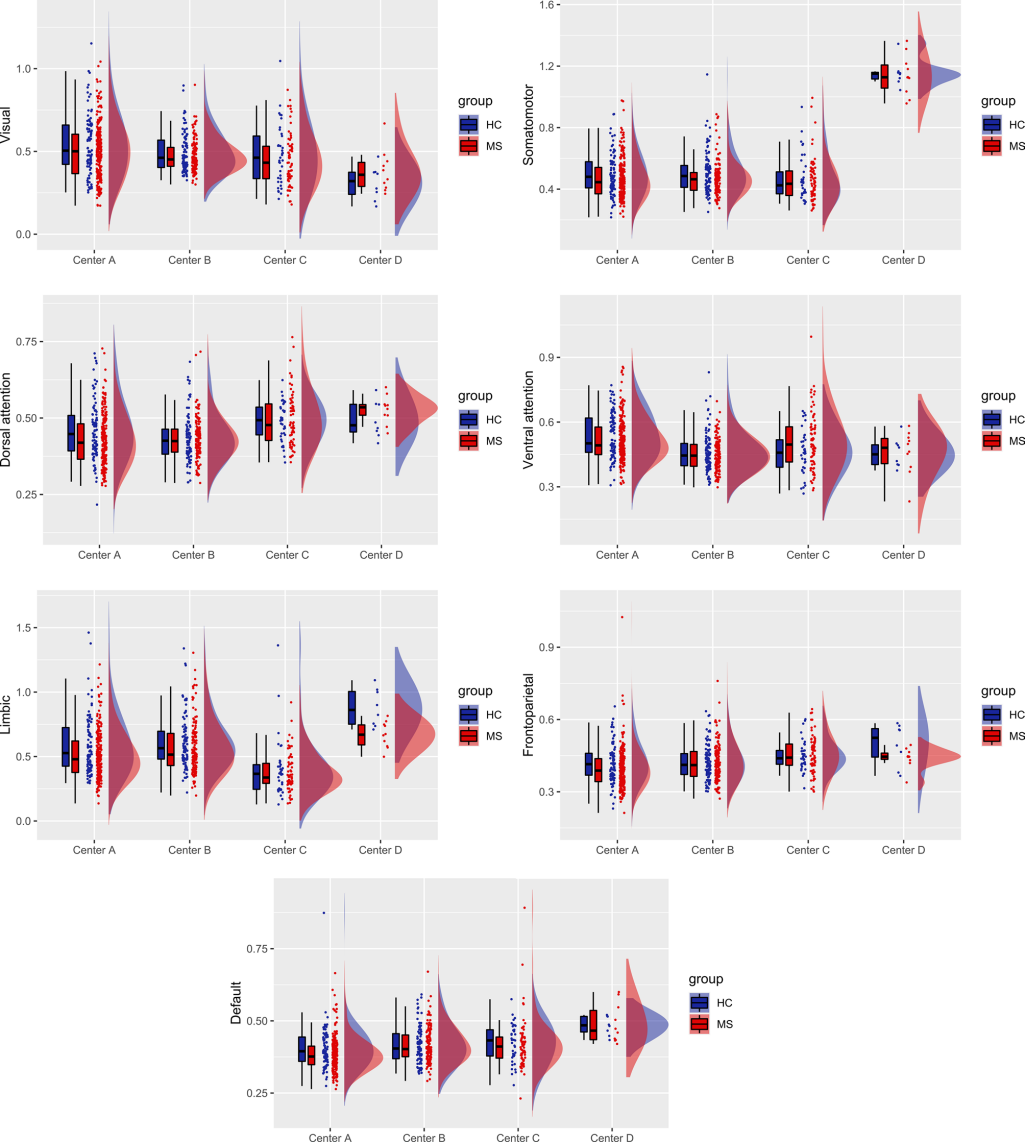
Boxplots and probability density estimation for the functional connectivity features in each of the seven functional networks from the Schaefer parcellation before ComBat harmonization (these plots were generated using the R package available at https://github.com/RainCloudPlots/RainCloudPlots). On the *y*-axis, data are reported as Fisher *z* transformed correlation coefficients

Figure 3 illustrates the boxplots and distributions for each center of the age-, sex- and mean FD-adjusted FC features (without harmonization) for the seven large-scale resting-state networks based on Yeo parcellation. There was a significant inter-site effect (*p* < 0.01) in each network and post hoc analyses revealed significant differences in most pair-wise comparisons between centers, with different networks showing different patterns of inter-site changes. Furthermore, when comparing the variance of the FC features across sites (only in HCs), the Fligner–Killeen test was significant for the VN (*p*-corrected < 0.01). From the pair-wise comparisons on the variance, Center A and Center B significantly differed in variance for the VN (*p* < 0.01).

**Fig. 4:**
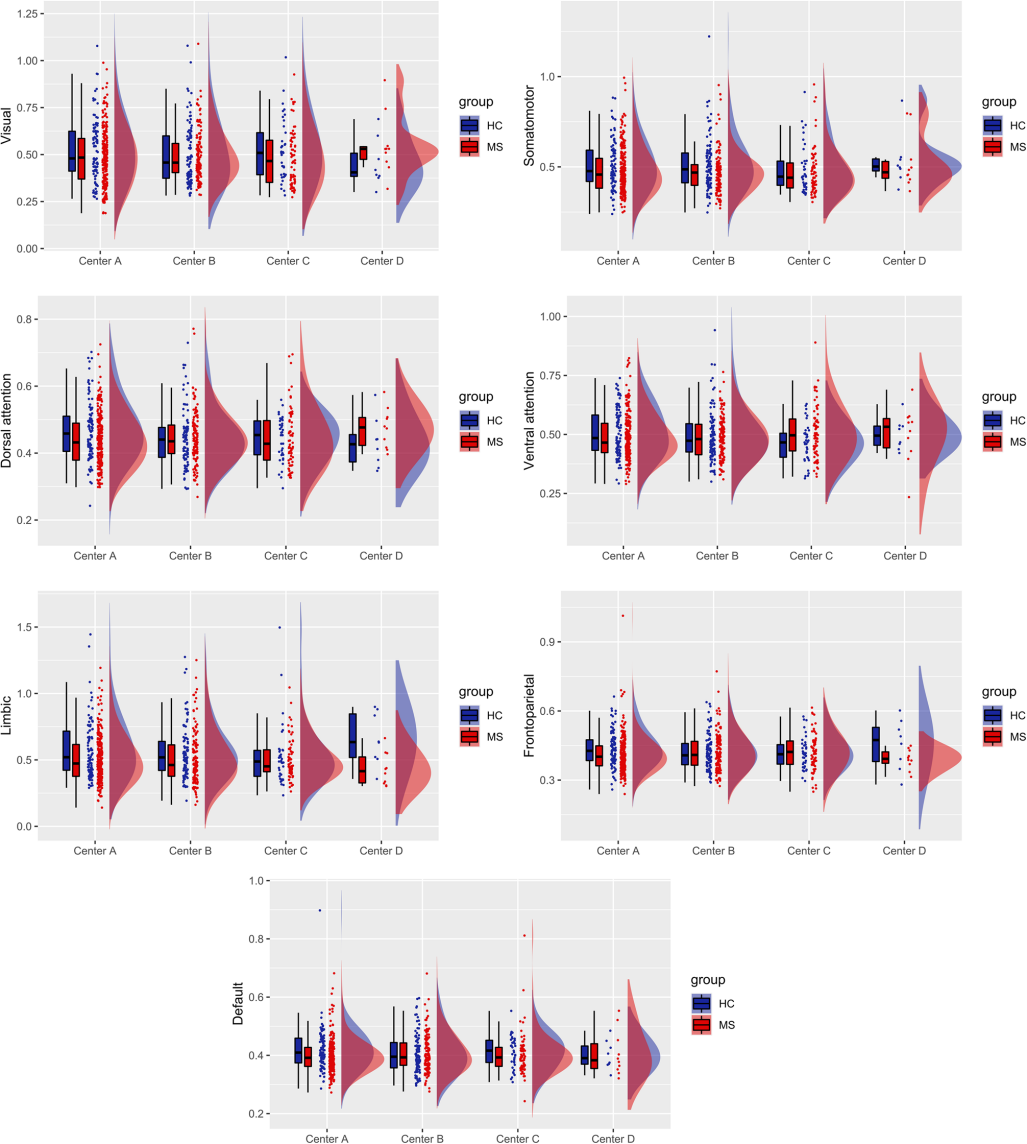
Boxplots and probability density estimation for the connectivity features in each of the seven functional networks from the Schaefer parcellation after ComBat harmonization (these plots were generated using the R package available at https://github.com/RainCloudPlots/RainCloudPlots). On the *y*-axis, values are reported as Fisher *z* transformed correlation coefficients

Figure 4 illustrates the boxplots and distributions for each center of the harmonized and age-, sex- and FD-adjusted FC features for the seven functional networks from Yeo parcellation. Expectedly, we did not find any residual inter-site effects in any of the seven networks. Also, Fligner–Killeen tests for variance homogeneity were not significant for any of the seven networks.

**Fig. 5:**
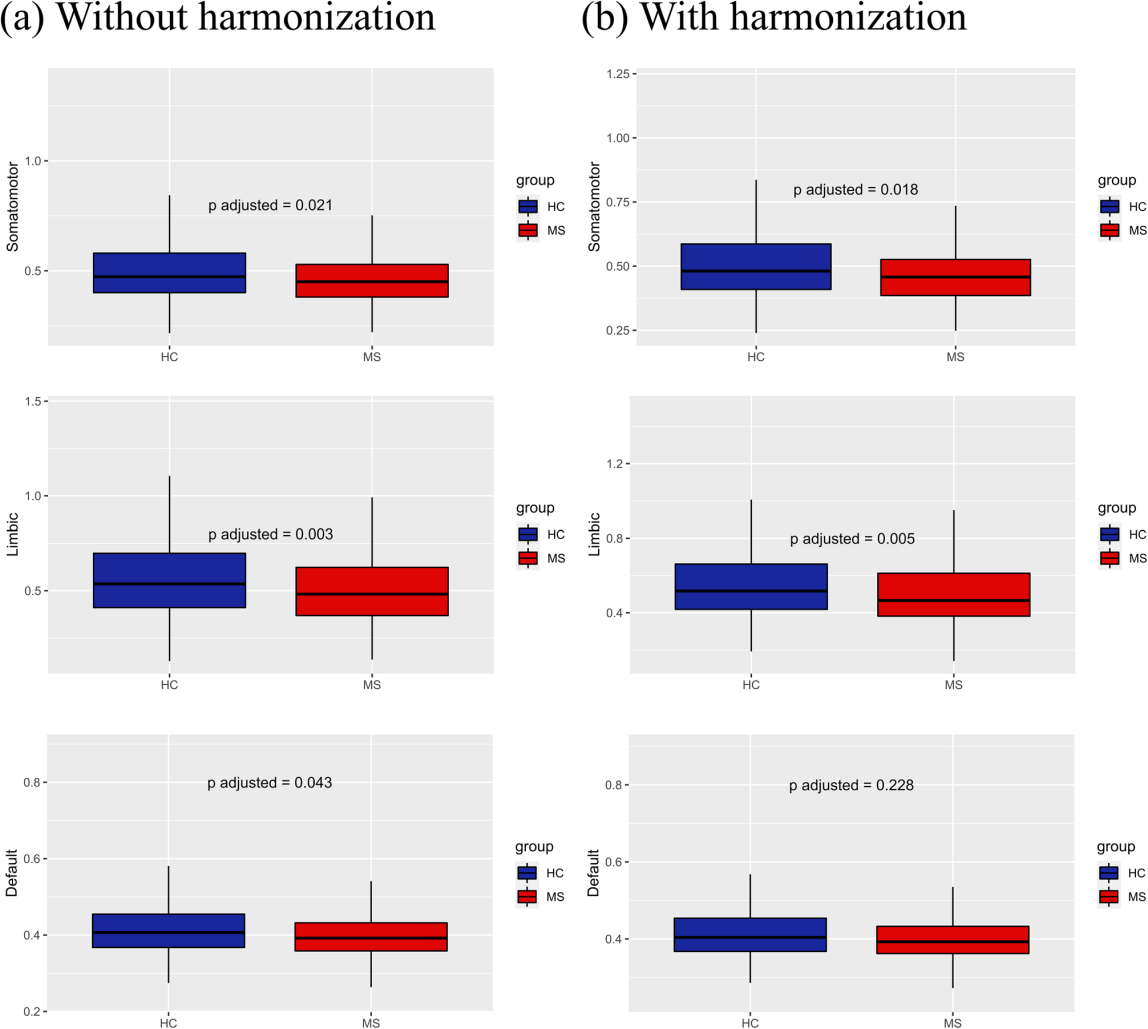
Resting-state networks showing statistically significant differences between MS and HCs without (**a**) and with ComBat harmonization (**b**). With feature harmonization statistical differences are preserved in SMN and LN, but not in DMN. On the *y*-axis, values are reported as Fisher *z* transformed correlation coefficients

At the level of groups, and without harmonization, MS patients showed a significantly decreased FC for the SMN (*p* < 0.01), the LN (*p* < 0.001), and the DMN (*p* < 0.05), as illustrated in the boxplots of Fig. 5a. After applying Combat harmonization, we found significant differences between MS patients and HCs across all centers for the SMN (*p* < 0.05) and the LN (*p* < 0.01) as illustrated in the boxplots of Fig. 5b. None of the comparisons between MS and HCs survived the Bonferroni correction for multiple comparisons in the within-center analyses, either with or without harmonization.

**Fig. 6:**
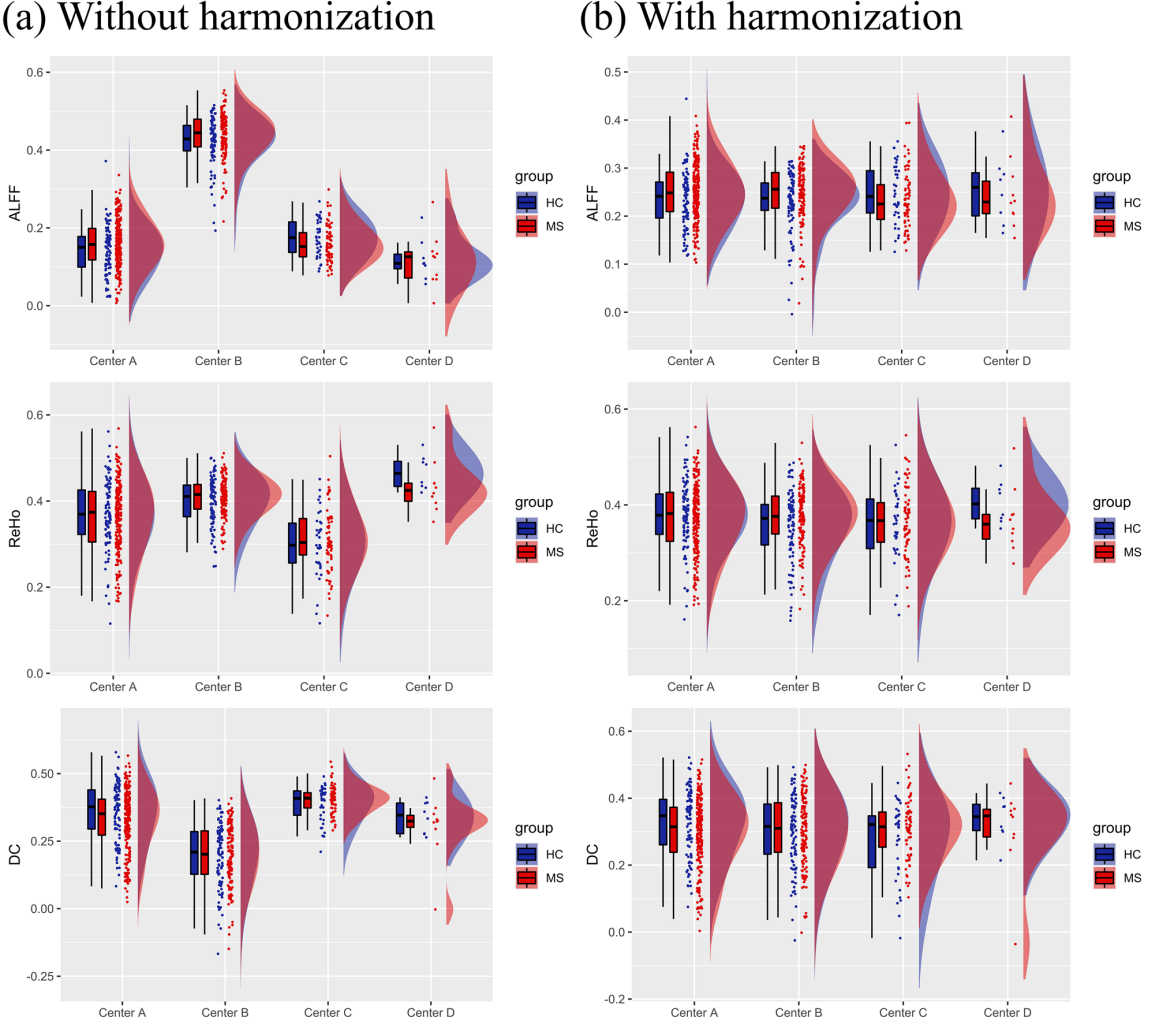
Boxplots and probability density estimation for the global connectivity features without (**a**) and with ComBat harmonization (**b**) (these plots were generated using the R package available at https://github.com/RainCloudPlots/RainCloudPlots)

**Fig. 7:**
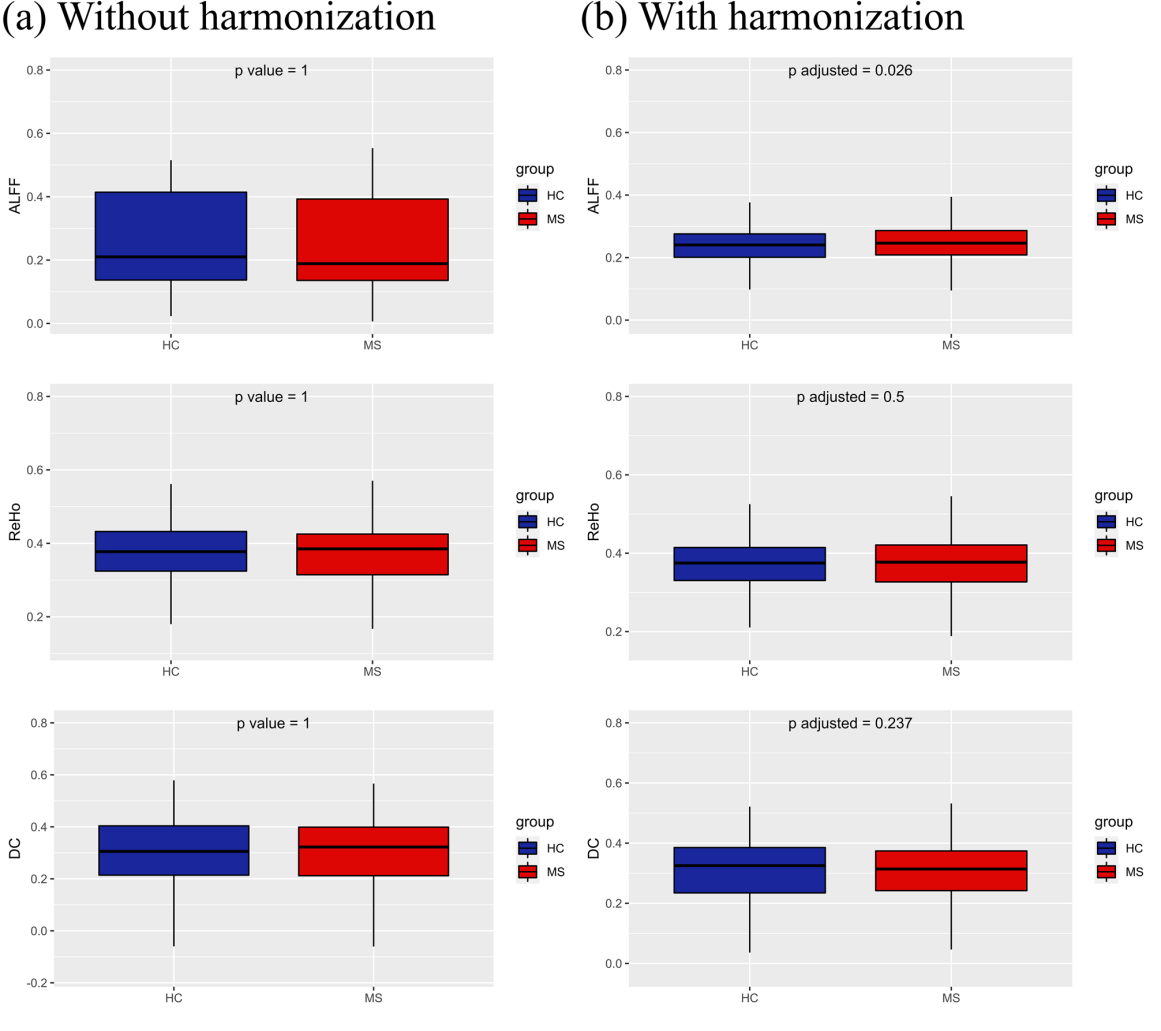
Boxplot of global connectivity features from pooled data without (**a**) and with ComBat harmonization (**b**)

Figure 6 illustrates the boxplots and distributions for the mean global features, as well as the corresponding metrics with harmonization applied. Before harmonization, all three global FC features (ALFF, ReHo and DC) exhibited significant differences between sites using different scanners. In the HC group, Fligner–Killeen test for variance homogeneity was significant only for DC (*p* < 0.01), with Center A and Center B showing more variability compared to Center C and Center D. At the level of groups, there were no significant differences between MS patients and HCs across all centers without harmonization (Fig. 7a), although within-center analyses revealed an increased ALFF in MS patients from Center B. After harmonization of global features, differences and variance heterogeneity (in HCs) were successfully removed. Group-level comparisons revealed that MS patients pooled from all centers showed a significant increase (*p* < 0.05) in ALFF, as illustrated in Fig. 7b. In within-center analysis, MS patients in Center B still showed an increased ALFF.

## Discussion

Controlling the quality and homogeneity of MRI datasets and MRI-derived features across different centers is a crucial prerequisite for obtaining reliable results in large multicenter brain MRI studies on MS [25]. In this work, we used validated IQMs to describe the quality and homogeneity of a representative sample of RS-fMRI data from the INNI database and investigated the extent to which inter-site effects impact the multisite analysis of some typical FC features after unified automated data preprocessing. This allows us to report the outcome of RS-fMRI data quality control for this database as well as to discuss the extent of inter-site effects emerging from the different acquisition hardware and procedures implemented in the four research centers affiliated with the INNI project. Ultimately, we provide important information to the repository for future multicenter studies on MS patients based on the available RS-fMRI data. Particularly, the presented characterization of RS-fMRI data currently included in the INNI database demonstrated that the collection of data from different centers (with different MRI scanners and acquisition protocols) poses specific challenges in terms of quality and homogeneity of the data.

The tSNR metric provides a convenient index for assessing the RS-fMRI time-course stability. In the literature, tSNR analyses have been often performed to compare acquisition protocols [56, 57], preprocessing pipelines [58, 59], and multisite datasets [60–63]. Within-site variability in tSNR values is essentially due to the variability in thermal and physiological noise sources [33]. Across sites, tSNR is expected to increase with increasing voxel size [64] and/or with an increasing number of time points in the series [59, 65]. Hence, we could possibly explain that Center C, which provides RS-fMRI acquisitions with only 140 time points (i.e., at least 60 time points less than the other three sites), had the lowest tSNR, and that Center B, which provides RS-fMRI acquisitions with 240 time points and the lowest resolution, had the highest tSNR. Inter-site differences in tSNR have been also related to the number of receive coils and the presence/absence of fat suppression [61]. Other studies have demonstrated how the presence or absence of different acceleration methods in EPI acquisition can affect the tSNR [33, 57]. Thus, we cannot exclude that the inter-site differences in tSNR could be partly explained by the different acceleration method.

FWHM provides a measure of the intrinsic spatial smoothness of the raw functional data and could therefore be related to the sensitivity of the RS-fMRI signals to the spatial characteristics of the underlying BOLD sources independently of the voxel size. Here, we observed statistically significant differences in the FWHM metric across sites that were not explained by voxel size as good as by the scanner type. In fact, we noticed that GE (Center B) and Philips scanners (centers A and D) exhibited greater FWHM values compared to a Siemens scanner (Center C), in line with previous works [66, 67]. A major cause of site differences in FWHM could be the presence (and type) or absence of spatial filtering during the image reconstruction process in k-space; in fact, Friedman and colleagues hypothesized in their work (a multicenter study where GE and Siemens scanners are included) that the key reason why images from GE scanners are smoother than those from Siemens scanners is the different k-space filtering algorithm employed [66]. Thus, the same reason could be attributed to explain the differences with Philips scanners. Given the importance of k-space filtering on raw smoothness, a better solution would be that all scanners use the same filter and the same filter settings. When this is not feasible, another strategy to possibly alleviate inter-site differences in FWHM would be the post hoc smoothness equalization, i.e., smoothing all images to a level equal to the largest FWHM estimated. According to Friedman et al. [66], spatial smoothing would also improve tSNR with beneficial effects on the overall ability to detect small changes in BOLD signal, although this increase would come at the price of decreased effective spatial resolution [66].

DVARS measures how much the intensity of the entire brain image varies in the comparison between two consecutive time points. Consequently, DVARS jointly indexes the amount of intra-voxel motion and physiological noise in the time series [30]. Since it is based on BOLD signal intensity, DVARS is expected to differ across different datasets, scanners and sequences, as indeed shown by results (Fig. 2c). Center C showed the lowest standardized DVARS values, thus indicating a lower level of global noise in fMRI data in comparison with other centers. Explaining inter-site differences in DVARS with the different technical parameters used in acquisition phase is not so straightforward, as this quality measure is likely influenced by inter-subject variability. This is potentially linked to the fact that DVARS is sensitive to temporal signal variations beyond those reflected in head motion, and thus might reflect some other source of artifactual signal variation, such as cardiac pulsation and respiratory rate variability [68].

Framewise displacement (FD) is the most typical metric to quantitatively assess the total amount of head motion that potentially corrupts RS-fMRI time-series. Indeed, FD is widely used in RS-fMRI studies to implement an exclusion criterion based on the amount of motion in the raw images prior to preprocessing (e.g., by setting a maximum threshold on the mean FD and/or on the proportion of time points with motion corrupted data as indexed by an instantaneous FD higher than a given threshold) or to implement various scrubbing strategies for motion artifact removal [29]. The exclusion of a small subset of high-motion datasets can dramatically improve the overall quality and reduce the correlation between motion and FC features [69]. As the effect of motion artifacts strongly depend on the compliance of the subjects, it could be expected that FD estimates are mostly independent from the MRI site, scanner and sequence [30], but this may not necessarily be the case, as showed in other works [70]. Indeed, we found a significant variability in the median FD across the four sites. In this case, such variability could be directly related to acquisition parameters. In fact, Center A and Center D, which showed the highest median FD, also had the smallest in-plane voxel dimensions (mm) in their acquisition protocol (1.875 × 1.875 × 4), whereas Center B and Center C had voxel dimensions, respectively, of 4 × 4 × 4 and 3 × 3 × 3. Thus, the different resolution of raw functional images (together with the isotropic voxel) also renders the data differentially sensitive to motion, as indexed by the FD metric. In addition, and not surprisingly, centers showing the highest median FD (A and D) also had the highest resting-state scan time (~ 600 s, against ~ 360 s and ~ 420 s for Center B and C, respectively). Indeed, shorter scan times obviously imply that patients are less likely to become uncomfortable and move during the same scan [71]. Moreover, a 32-channel coil was used in Center D, thereby we could expect an even higher sensitivity to motion events from the acquisitions in Center D. Ideally, the impact of motion on pre-processed data should be minimal after proper correction; however, residual motion-related effects might still affect the data variably across patients, thereby regressing out the mean FD from the variance of FC features is generally advised [72].

Overall, for the IQMs considered here, we did not expect to find any significant difference between populations within each site (considering that all subjects within a site were acquired with the same scanner and RS-fMRI protocol). However, surprisingly, we found a significant difference (*p* < 0.01, after correction) in FWHM between MS patients and HCs for Center A. As stated before, FWHM measures the degree of inherent smoothness of an image, thus the higher the degree of spatial homogeneity of the image, the higher the FWHM. In addition, longer repetition times improve tissue contrast in EPI images [73]. Thus, we can hypothesize that lower tissue contrast plays a role in increased FWHM. This would explain why in Center B (TR = 1.5 s), FWHM values are higher compared to all other centers (TR = 3 s). Because signals of neural origin are not present in CSF (and WM), this compartment (whose signal reflects only physiological and hardware noise) could largely contribute to the overall image smoothness. Subsequently, the absence of significant difference between patients and controls in other sites could be at least partially explained by two factors: first, we found that only in Center A (*p* < 0.01, after correction) and in Center B (*p* < 0.05, after correction), MS patients had greater CSF volume compared to controls (see Table 3), meaning that only in these two centers, we could allegedly expect some difference between groups; second, the lack of any difference in Center B could be explained by the lower tissue contrast that would mitigate the contribute of the CSF to the FWHM values.

Contrary to tSNR and FWHM, which were reported in our QC procedure merely as descriptive measures of the data, DVARS and FD were also used to investigate how residual physiological noise and motion differently affect FC features in each center after preprocessing. Thus, to possibly address the actual (residual) dependence of all FC features from the amount of noise and motion, we also considered the site-wise correlations between mean FD and DVARS (estimated prior to preprocessing) and all FC features (after preprocessing), after regressing out age and sex. For those sites for which such correlations would be statistically significant (i.e., *p* < 0.05 after correction for multiple sites), we could eventually draw an indication that noisy data are still influencing the FC features, even after denoising. However, overall, our results seem to indicate that the variance of residual head motion (accounted by FD and DVARS) does not greatly contribute to the variance fMRI-derived network features, suggesting that the chosen preprocessing pipeline did not introduce an extra-bias (e.g., due to filtering and shaping of the data) because of the technical differences between sites (also noting that in Center D, the importance of higher *R*^2^ values is hampered by the small-sample size [74, 75]. Nonetheless, global features like ALFF, ReHo, and DC seemed to be more influenced by DVARS (both in Center A and Center B) and head motion (only in Center B). Motion and physiological noise (especially in regions near blood vessels and ventricles) have already been found to have an impact on ALFF [69, 76]. ReHo, as a measure of local connectivity, may be particularly sensitive to cardiac and respiration effects [77]. As of DC, it is known that physiological noise can also result in higher correlations between brain regions [78], thus possibly contributing to influence DC values. Our results suggest that our unified automated pipeline successfully removed noise- and motion-related variability from network-derived features in all centers. However, global maps calculated in a few sites were (differently) influenced by global noise, thus suggesting that caution should be taken when using these features: for example, including mean FD as covariate to regress (as we did in our subsequent analyses) is probably in order and might be beneficial.

After proper exclusion of high-motion subjects, to possibly address site-related differences after preprocessing, we first analyzed any possible site-related effect on the most typical and widely reported FC measures as derived from pre-processed RS-fMRI signals using Pearson correlation statistics and a standard network parcellation of the cerebral cortex, after regressing out age, sex, and mean FD for each site. As we used a 100-parcel atlas, the reproducibility of these results might also depend on the specific parcellation size adopted in the unified RS-fMRI data processing pipeline. Indeed, Yu et al. showed that the magnitude of site-related effects in RS-fMRI functional connectivity analyses is not invariant to the parcellation size [79]. For example, one might expect that when the effect of interest is highly focal, such as, e.g., in the case of a seed-based functional connectivity analysis from a specific region to the whole brain, increasing the size of the parcellation might be more effective in terms of reproducibility of the metric. However, when the focus is on the analysis of large-scale functional connectivity networks, as is the case for the present work, reducing the size of the parcellation should be preferrable [41]. Of note, as most of the resting-state networks can be variably altered in MS pathology [80], e.g., due to phenotype, therapy, symptoms, and immunomodulatory, it should be expected that, besides the site effects discussed here, the MS clinical variability of the samples across sites would contribute to the inter/intra-site variability. Prior to harmonization, we found that the FC was significantly different across the four sites in all networks. However, variance across sites was quite homogeneous in the control groups, with the only exception of the visual network. Site effects in multisite RS-fMRI measurements have been widely reported in previous studies [52, 79, 81]. Not differently from other multisite datasets, in the INNI repository, the consistent inter-site variability in the RS-fMRI measurements may be attributed mainly to differences in imaging protocol parameters [82]. These differences could be standardized to some extent, but site effects will still be unavoidable in large multicenter studies [52]. In fact, other sources of variability in RS-fMRI connectivity can be attributed to differences in clinical and demographical characteristics. However, regression-based statistical procedures can be performed to model and, subsequently, remove unwanted site effects. Here, we considered using the ComBat harmonization algorithm, which was previously developed and used in genomics [83], and recently adapted and successfully applied to multicenter MRI datasets [53, 54, 79].

To assess whether site effects remained after harmonization, we repeated the same statistical analyses. We found that ComBat harmonization successfully corrected for unwanted site effects in all networks. Moreover, ComBat was also able to remove the scaling effects associated with site in the visual network, proving also its usefulness to homogenize variances across different groups. After harmonization, pooled MS patients from all centers showed reduced within-network FC in somatomotor and limbic networks. Abnormalities of FC within these two networks were reported in previous studies using a variety of scanners and methods [84–86]. Of note, from pooled non-harmonized data, we found significant differences also in default-mode network. Since this difference did not arise from pooled harmonized data, it is very likely that it was driven by inter-site effects. On the contrary, the differences in the SMN and LN were still present after harmonization, reflecting a possible actual alteration in MS FC.

Site-related effects were also observed for global features, such as ALFF, ReHo and DC, after averaging over the entire gray matter. However, this was not surprising, as calculation of these features can be easily impacted by different acquisition parameters.

In a previous work, significant differences in ALFF emerged between Siemens and GE sites, with the latter showing a higher mean value [87], and this was consistently observed in our study. In Wang et al., extremely large site-related effects for ALFF were mainly attributed to signal scaling, as BOLD signal has arbitrary units and is often scaled dissimilarly across different MRI scanners [88]. However, the signal scaling effect should not intervene after standardization, whereas other factors may cause site-related effects of ALFF, such as different TRs. This would explain why the only site showing significant variability against all other sites was Center B, i.e., the only one with a different TR. On the other hand, sites using the same TR (Center A, Center C, and Center D) showed no differences in ALFF values.

ReHo is computed with Kendall’s Coefficient of Concordance (KCC), which is an index that depends on the time series of each voxel, the number of neighboring voxels (set to 26), and the number of time points. This last variable can explain why Center C (140 time points) showed the lowest ReHo values, whereas Center B (240 time points) showed the highest ReHo. On this basis, we expected that sites using the same number of time points (centers A and D), i.e., 200 volumes, would not display significant variability in ReHo values, as indeed shown by the results. In addition, it should be also noted that the native voxel resolution of the data from centers A and D is quite higher than the native voxel resolution of centers B and C, thereby a lower impact was to be expected for centers A and D for the interpolation needed to bring data from native to MNI voxel space, compared to centers B and C. However, while different degrees of interpolation necessarily cause different contributions of the signals from the original (native) voxels at each individual location, the global metric derived here from the ReHo maps is calculated from the spatial averaging of all GM voxels, thereby the impact of the interpolation on the global metric is expectedly reduced.

Finally, DC is a measure that counts the number of voxels correlated with a target voxel. Having calculated it directly in MNI152, differences between sites cannot be justified with the different voxel sizes. Instead, we suppose there is an inverse relationship of degree centrality values with the number of time points. As shown by results, Center C (140 time points) had the highest DC values, and Center B (240 time points) had the lowest DC values. This could be explained hypothesizing that a lower number of volumes contribute to increase the number of voxels with spurious correlation above threshold.

## Conclusions

In conclusion, the QC analysis performed in this study provides a systematic quality assessment of RS-fMRI data collected from MS patients and HCs within the INNI repository, with the goal of promoting and implementing procedures for a more harmonized use of fMRI data and to provide reference information and initial guidelines for the design of large-scale harmonized fMRI studies in MS. Our proposed automated pipeline, characterized by the implementation of a unified preprocessing workflow for pooled multisite fMRI data, introduced little or no bias in FC features, making it a valuable tool for future multisite studies in INNI. In fact, both the correlations between pre-parcellated brain regions and the distribution of the most popular metrics of spontaneous brain activity exhibited site-related effects that were successfully removed with a simple statistical harmonization procedure, ultimately exhibiting an acceptable level of variance homogeneity in the control group and a good consistency of the inter-group effects. Nonetheless, this work is merely descriptive of a subset of the subjects included in the INNI repository and concerns regarding the within-subject reproducibility of the RS-fMRI metrics have been raised [89]. Consequently, the lack of a within-subject reproducibility analysis represents a limitation of this study and future QC studies within INNI should possibly address this aspect, e.g., by adding repeated RS-fMRI acquisitions in the protocol. As a full standardization of fMRI acquisition protocols across sites was not requested in the first phase of the INNI project [16], more can be done in the acquisition phase to reduce variability across sites and improve robustness and reproducibility of quantitative fMRI measures. Moreover, based on our results, it will be highly recommendable to harmonize fMRI-derived measures to minimize site-related effects, while preserving inter-subject biological variability.
