# Correction to: Resting-state functional MRI in multicenter studies on multiple sclerosis: a report on raw data quality and functional connectivity features from the Italian Neuroimaging Network Initiative

**DOI:** 10.1007/s00415-023-11646-w

**Published:** 2023-03-13

**Authors:** Alessandro Pasquale De Rosa, Fabrizio Esposito, Paola Valsasina, Alessandro d’Ambrosio, Alvino Bisecco, Maria A. Rocca, Silvia Tommasin, Chiara Marzi, Nicola De Stefano, Marco Battaglini, Patrizia Pantano, Mario Cirillo, Gioacchino Tedeschi, Massimo Filippi, Antonio Gallo, the INNI Network, Manuela Altieri, Riccardo Borgo, Rocco Capuano, Loredana Storelli, Elisabetta Pagani, Mauro Sibilia, Claudia Piervincenzi, Serena Ruggieri, Nikolaos Petsas, Rosa Cortese, Maria Laura Stromillo

**Affiliations:** 1 Department of Advanced Medical and Surgical Sciences, University of Campania “Luigi Vanvitelli”, Piazza Luigi Miraglia, 2, 80138 Naples, Italy; 2 Neuroimaging Research Unit, Division of Neuroscience, IRCCS San Raffaele Scientific Institute, Via Olgettina 60, 20132 Milan, Italy; 3 Neurology Unit, IRCCS San Raffaele Scientific Institute, Via Olgettina 60, 20132 Milan, Italy; 4 Neurorehabilitation Unit, IRCCS San Raffaele Scientific Institute, Via Olgettina 60, 20132 Milan, Italy; 5 Neurophysiology Service, IRCCS San Raffaele Scientific Institute, Via Olgettina 60, 20132 Milan, Italy; 6 Vita-Salute San Raffaele University, Via Olgettina 58, 20132 Milan, Italy; 7 Department of Human Neurosciences, Sapienza University of Rome, Viale Dell’Università, 30, 00185 Rome, Italy; 8 Institute of Applied Physics “Nello Cararra” (IFAC), National Research Council (CNR), Via Madonna del Piano, 10, Sesto Fiorentino, 50019 Florence, Italy; 9 Department of Medicine, Surgery and Neuroscience, University of Siena, Siena, Italy; 10 IRCCS Neuromed, Pozzilli, IS Italy

**Correction to: Journal of Neurology (2023) 270:1047–1066** 10.1007/s00415-022-11479-z

The original version of this article unfortunately contained a mistake. The second affiliation for author "Patrizia Pantano” was missing.

The second affiliation should be

IRCCS Neuromed, Pozzilli (IS), Italy.

